# Do Serum Vitamin D Levels Have Any Effect on Intrauterine Insemination Success? 

**DOI:** 10.22074/ijfs.2018.5256

**Published:** 2018-03-18

**Authors:** Nafiye Yilmaz, Ebru Ersoy, Aytekin Tokmak, Ayla Sargin, A. Seval Ozgu-Erdinc, Salim Erkaya, Halil Ibrahim Yakut

**Affiliations:** 1 Department of Obstetrics and Gynaecology, Zekai Tahir Burak Women’s Healthcare Training and Research Hospital, Ankara, Turkey; 2 Zekai Tahir Burak Women’s Healthcare Training and Research Hospital, Ankara, Turkey

**Keywords:** Infertility, Intrauterine Insemination, Ovulation Induction, Vitamin D

## Abstract

**Background:**

Recent studies have shown that vitamin D has an essential role in the reproductive system. In this study, we aimed to investigate the effect of vitamin D levels in patients undergoing ovulation induction (OI), and subsequent intrauterine insemination (IUI) procedure.

**Materials and Methods:**

One hundred and four infertile and one hundred and three fertile women were recruited in this cross-sectional study which was conducted in a tertiary level maternity hospital. Infertile patients were divided into pregnant and non-pregnant subgroups after treatment. Individual characteristics and 25-hydroxyvitamin D_3_[25 (OH) D_3_] levels were compared between the groups.

**Results:**

The vast majority of our study population consisted of women who had vitamin D deficiency (96.6%). There was no statistically significant difference between infertile and fertile groups in terms of serum 25 (OH) D_3_lev- els (P=0.512). Similarly, no significant difference was observed between the pregnant and non-pregnant subgroups of infertile patients regarding 25 (OH) D_3_levels (P=0.267).

**Conclusion:**

There is no association between female infertility and serum vitamin D levels. Vitamin D does not pre- dict pregnancy in infertile women undergoing OI with IUI. Further research which will provide a comparison between much more women who have deficient and sufficient 25 (OH) D_3_levels is warranted.

## Introduction

Intrauterine insemination (IUI) is used to transport spermdirectly into the uterus. It is a simple, non-invasive, andcost-effective technique used for assisted reproduction. Themost common indication for IUI is cervical infertility, and it is also used in male subfertility, anovulation, endometriosis cases in which at least one tube is healthy, as well as unexplained infertility (1, 2). Although there may be a trendtowards higher pregnancy rates when the number of IUIsper cycle is increased, a recent meta-analysis has shownthat increased IUI numbers do not increase pregnancy (3). Previous investigations reported that IUI had a success rateof 10-20% for clinical pregnancies (4).

Recently, the effect of vitamin D (VD) has been investigated on not only the musculoskeletal system, but also inthe reproductive and other systems (5). The biologic actionsof VD are mediated through the vitamin D receptor (VDR). VDR was found to be in the ovary (particularly the granulosacells), uterus, placenta, and testis, suggesting VD may have a significant role in human reproduction (6). Two studies supporting this data indicated that VD deficiency is responsible for reduced fertility and reproductive capacity in female rats (7, 8). Research conducted on human subjects also supports this role as in experimental animal studies (9).

Calcitriol (1, 25 dihydroxyvitamin D_3_) which is the active form of VD stimulates CYP19 expression (CYP19 encodes the aromatase enzyme) that results in increased estrogen production, when it was bound to VDR (10). Furthermore, it has been reported that decidua secretes calcitriol during blastocyst implantation, and calcitriol has been reported to regulate the immune response in the maternal-fetal interface during pregnancy (11).

There are several studies which presented controversial results on the differences in 25-hydroxyvitamin D_3_[25 (OH) D_3_] levels of the patients undergoing different infertility treatment modalities (12, 13). The aim of our study was to investigate the 25 (OH) D_3_ levels in patients who underwent ovulation induction with IUI and then to determine the relationship between 25 (OH) D_3_ levels and the occurrence of pregnancy.

## Materials and Methods

This case-control study was conducted between March 2014 and June 2014 in the infertility outpatient clinics of Zekai Tahir Burak Women’s Health Education and Research Hospital. This is a government supported tertiary level maternity hospital located in the capital city of Turkey. The institutional review board approved the study and informed consent was obtained from each patient (approval number: 23.09.2013/9). All of the study protocols were carried out in accordance with the Helsinki Decleration (14). 

We defined the infertile patients as those reproductive age couples who were unable to become pregnant in the absence of contraception. For the women below 35 years of age, infertility was diagnosed as a minimum of 1 year of trying to become pregnant, whilst for the women above 35 years of age, the diagnosis was limited to 6 months of unprotected sexual intercourse. After we obtained detailed information about age, duration of infertility, infertility type, previous history of surgery, and any systemic disturbances (such as diabetes mellitus, hypertension, and thyroidal disease), a complete physical and gynaecological examination was performed on all of the women. We confirmed tubal patency in the women using hysterosalpingography (HSG) and if there was bilateral tubal occlusion detected with HSG, we applied laparoscopy and hysteroscopy to define any pathology such as pelvic adhesions or endometriosis. When we suspected an intracavitary lesion in the uterus after HSG, or transvaginal ultrasound, we performed hysteroscopy. 

We included women with mild male factor infertility, unexplained infertility, and polycystic ovary syndrome (PCOS). We excluded patients who had advanced age (above 40 years of age), any systemic or endocrine diseases, stage 3-4 endometriosis, or intracavitary lesions in uterus (such as endometrial polyp, submucous myoma, and uterine septum), smokers and women who used of any kinds of drugs or substances likely to affect levels of VD. We also excluded patients whose partner had a motile sperm count lower than 5 million/mL. The fertile group consisted of patients who applied to the family planning unit of our hospital for contraceptions. These patients had given birth in the previous 12 months, has not breastfed their neonate, and had no history of infertility.

After initial clinical assessment, infertile patients were evaluated for clomiphene citrate (CC) or gonadotropins (Gn) and IUI use. Those patients who had used CC with IUI treatments for three times or were above 35 years of age were directed into the Gn with IUI regimen (n=63), whilst the other infertile patients were directed into the CC and IUI regimen (n=41). When 18-20 mm (dominant follicles) were found through ultrasound, 2 human chorionic gonadotropin (hCG, Pregnyl, MSD, Netherlands) ampoules containing 5,000 units each, were injected intramuscularly, and IUI applied 36 hours after the injection. When there were 3 or more dominant follicles, or endometrial thickness was less than 6 mm, hCG was not administered. Then 2 weeks later, a blood sample was obtained from patients for ß-hCG measurement. Clinical pregnancy was diagnosed 5 weeks after IUI, when the evidence of fetal heart activity or presence of the gestational sac in the uterine cavity was detected.

The concentration of serum 25 (OH) D_3_ was used to determine the status of VD in the body for this study since it has been proven to be the best biomarker for VD insufficiency. It also reflects VD levels from both dietary intake and in-skin synthesis (6). The two groups were matched in term of veiling habits, daily exposure to sunlight, and dietary intake of VD-rich foods which was determined by a dietician.

The serum levels of 25 (OH) D_3_ levels and baseline hormones including estradiol, follicle stimulating hormone (FSH), luteinizing hormone, prolactin, and thyroid stimulating hormone were measured on the third day of the menstrual cycle when ovulation induction was started. We performed the recruitment of study volunteers in a single season, because the blood levels of VD have seasonal variabilities (15, 16). In addition, patients living in the same geographical region were selected for the study (17).

After overnight fasting, venous blood samples were obtained early in the morning and transferred to the laboratory in a non-transparant box to avoid exposure to light, and then serum was separated by centrifugation at 5,000 rpm (2,236 g) for 10 minutes. The serum 25 (OH) D_3_ levels were measured using an enzyme linked immunosorbent assay kit (Immunodiagnostic AG, Leverkusen, Germany), and presented in ng/mL. The intra-assay and inter-assay coefficients of variation for serum 25 (OH) D_3_, were 8.9 and 10.6% respectively. Serum 25 (OH) D_3_ concentrations <20 ng/mL was considered as VD deficiency. Types of VD deficiency were also classified as mild (10-20 ng/ mL), moderate (5-10 ng/mL), and severe (<5 ng/mL). Serum 25 (OH) D_3_ concentrations between 20 and 30 ng/ mL was accepted as VD insufficiency whereas a threshold value of =30 ng/mL was considered sufficient serum VD levels. Basal hormone levels were measured using an Immulite 2000 analyzer (EURO/DPC Ltd., Gwynedd, UK). Body mass index (BMI) was defined as the weight in kilograms divided by the square of the height in meters.

We examined the women who had a positive result for ß-hCG using transvaginal ultrasound at at least weeks 6-7 of gestation to detect fetal cardiac activity. The difference between the two subgroups (pregnant and non-pregnant) of infertile patients in terms of 25 (OH) D_3_ levels was the primary outcome measured of this study. The secondary outcome was the comparison of serum 25 (OH) D_3_ levels between infertile and fertile groups.

Data were recorded and analysed using the Statistical Package for the Social Sciences program for Windows version 17.0 (SPSS Inc, Chicago, IL, USA). The normal distribution of the variables was assessed using the Shapiro- Wilk’s test. Continuous variables were presented as the mean with standard deviation (SD) or median (range), and categorical variables were presented as the number (percentage) of subjects. Continuous variables were compared using independent samples t-test if they were normally distributed or with the Mann-Whitney U test if they were non-normally distributed. Categorical variables were analyzed using the Chi-square (χ^2^) test or Fisher’s exact test. Correlations were calculated using Spearman's correlation analysis. In all analyses, two-tailed P<0.05 were considered as statistically significant. Post-hoc power analysis demonstrated that we achieved a power of 0.95 with a 5% level of significance and a 0.5 effect size by using a two sample comparison (18). Power analysis was carried out on G-power software (G-power v3.1.9.2, Universitat Kiel, Kiel, Germany). 

## Results

One hundred and four infertile and one hundred and three fertile women were included into this cross-sectional, case-control study. Examination of the infertile and fertile patients showed that there was no statistically significant difference between the groups regarding their mean age and BMI. Obstetric history characteristics were statistically significantly different between the two groups (P<0.001 for all). The mean FSH levels were higher in the infertile patients than in the fertile patients (7.4 ± 2.1 mU/mL vs 6.2 ± 1.6 mU/mL, P=0.001), but it was within the normal range in either group. Mean prolactin levels of the fertile group (14.4 ± 5.4 ng/mL) were higher than the infertile group’s (12.2 ± 4.6 ng/mL). This difference was statistically meaningful (P=0.002), but those values were in the normal range as with mean FSH levels. There were no statistically significant differences in 25 (OH) D_3_ levels between the 2 groups [7.3 (3-25.5) ng/mL vs. 6.8 (3.4-37.1) ng/mL, P=0.512], as seen in Table 1. No significant correlation between serum 25 (OH) D_3_ and FSH levels was observed either in the entire study population (Spearman’s r=0.051, P=0.466).

**Table 1:** Descriptive characteristics and serum 25 (OH) D_3_ levels of infertile and fertile patients


Characteristic	Infertile group n=104	Fertile group n=103	P value

Age (Y)^**^	28.1 (4.7)	29.4 (5.4)	0.088^a^
BMI (kg/m^2^)^**^	25.1 (3.6)	25.7 (3.8)	0.234^a^
Gravida^*^	0 (0-6)	2 (1-6)	<0.001^b^
Parity^*^	0 (0-1)	2 (0-5)	<0.001^b^
Alive^*^	0 (0-1)	2 (0-5)	<0.001^b^
Abortion^*^	0 (0-5)	0 (0-4)	<0.001^b^
FSH (mIU/mL)^**^	7.4 (2.1)	6.2 (1.6)	0.001^a^
LH (mIU/mL)^**^	5.3 (2.5)	4.9 (1.9)	0.154^a^
Estradiol (pg/mL)^*^	44.0 (12-148)	42.7 (21-99)	0.791^b^
TSH (µlU/mL)^**^	1.9 (0.8)	2 (0.9)	0.859^a^
Prolactin (ng/mL)^**^	12.2 (4.6)	14.4 (5.4)	0.002^a^
25 (OH) D_3_ (ng/mL)^*^	7.3 (3-25.5)	6.8 (3.4-37.1)	0.512^b^


BMI; Body mass index, FSH; Follicle-stimulating hormone, LH; Luteinizing hormone, TSH; Thyroid stimulating hormone, *; Median (minimum-maximum), **; Mean (SD), ^a^; Student t Test, and ^b^; Mann Whitney U test. P<0.05 is considered as statistically significant.

The severity of VD deficiency in the infertile and fertile groups showed that most of the participants were deficient for VD (96.2 vs. 97.1%) and only 1 (1%) participant in the fertile group had 25 (OH) D_3_ levels =30 ng/mL. 19 (18.3%) patients were in the severe deficiency group, 56 (53.8%) cases were in the moderate deficiency group, and lastly 25 (24%) women were in the mild deficiency group among the infertile group. The number of fertile patients in the same groups was 19 (18.4%), 58 (56.3%), and 23 (22.3%), respectively (P=0.776). 

After IUI treatment, the numbers of clinical pregnancies and live births among 104 infertile patients were 14 (13.3%) and 10 (9.61%), respectively. When infertile patients were divided into two subgroups (pregnant and non- pregnant), there was no statistically significant difference between these subgroups regarding age, BMI, obstetrical history, baseline hormone levels, or ovulation induction agent used. Similarly, no significant difference was observed between the pregnant and non-pregnant subgroups of infertile patients in terms of serum 25 (OH) D_3_ levels (P=0.267). Ten (71.4%) patients out of the 14 clinical pregnancies had moderately deficient VD levels. The only significant parameter that may predict pregnancy was the age of the patients, namely the pregnant group was statistically significantly younger than the non-pregnant group (Table 2).

**Table 2:** Individual characteristics, ovulation induction type and vitamin D levels in pregnant and non-pregnant patients after IUI


Characteristic		Non-pregnantgroup n=90	Pregnant groupn=14	P value

Age (Y)^**^		28.5 (4.7)	25.5 (4.4)	0.027^a^
BMI (kg/m^2^)^**^		25 (3.5)	25.3 (4)	0.784^a^
Gravida^*^		0 (0-6)	0 (0-2)	0.745^b^
Parity^*^		0 (0-1)	0 (0-1)	0.459^b^
Alive^*^		0 (0-1)	0 (0-1)	0.459^b^
Miscarriage^*^		0 (0-5)	0 (0-2)	0.335^b^
FSH (mIU/ml)^**^		7.1 (3.5-13.6)	6.8 (3.4-13.5)	0.378^a^
LH (mIU/ml)^**^		4.9 (2.1-14)	5.9 (2.2-10.3)	0.247^a^
Estradiol (pg/ml)^**^		44.5 (12-148)	42.5 (20-82)	0.398^a^
TSH (µlU/ml)^**^		1.8 (0.4-5.3)	2.0 (1.3-3.5)	0.160^a^
Prolactin (ng/ml)^**^		12.3 (4.9-28.4)	12.9 (7.6-20.9)	0.788^a^
25 (OH) D_3_ (ng/mL)^*^		7.3 (3-25.5)	8.1 (4.7-22.1)	0.267^b^
Ovulation induction type^***^				0.777^c^
	CC	55 (61.1)	8 (57.1)	
	Gn	35 (38.9)	6 (42.9)	


IUI; Intrauterine insemination, BMI; Body mass index, FSH; Follicle-stimulating hormone, LH; Luteinizing hormone, TSH; Thyroid stimulating hormone, CC; Ovulation induction with clomiphene citrate, Gn; Ovulation induction with gonadotropin, *; Median (minimum-maximum), **; Mean (SD), ***; n (%), ^a^; Student’s t test, ^b^; Mann-Whitney U test, and ^c^; Fisher’s exact test. P<0.05 is considered as statistically significant.

## Discussion

Our study showed that infertile and fertile patients had similar serum VD levels and that there was no statistically significant difference in serum VD measurements between the pregnant and non-pregnant groups after IUI.

VD has an essential role in both male and female reproductive system (19). It was found that its deficiency is highly prevalent among women undergoing ovarian stimulation (9). Considering the previous data, we designed such a study assuming that VD could be lower in infertile patients, but we found no relationship between them. This result may be due to the fact that VD deficiency is very common in our study population, because 200 of the 207 patients also including women with no fertility problem had VD deficiency at the initial examination. This was a surprise and suggests that fertile patients who had a delivery in the preceding 12 months may have exhausted their VD stores during the most recent pregnancy and they had not been able to replace it yet. VD deficiency is one of the general public health matters in our country, with similar inferences having been suggested in other studies from our country (20, 21).

Ovulation induction with IUI is the most utilized method of infertility treatment in our unit. The success of IUI treatment is multifactorial, and pregnancy rates per cycle have been estimated as 10.2% in a IUI cycle with controlled ovarian stimulation (22).

A study by Ott et al. (23) demonstrated that 25 (OH) D_3_ levels may predict ovarian response to ovarian stimulation. This suggestion is consistent with another study showing that VDR exists in human ovaries and is important for sex steroid synthesis (10). However, when we compared pregnant patients with non pregnant patients in terms of serum VD levels, there was no statistically significant difference between them. This may be associated to the differences of individual VDR receptivity and VDR polymorphism (24). Although VDR polymorphism has been reported as not being related to infertility in an endometriosis study, there is a need for further research to clarify this particular issue (25). Another noteworthy result of our study is that patients who became pregnant after IUI treatment were younger than those who did not. It has already been shown that age is one of the most important factors in infertility management (26).

In two rat studies, VD deficiency was shown to significantly increase infertility, decrease probability of viable births and healthy full-grown individuals (8, 27). Although the exact mechanism remains to be elucidated, compromised ovarian folliculogenesis and infertility were found in two studies conducted on VD deficient mice (28, 29). A recent human study supporting these inferences showed that there is a negative correlation between serum levels of 25 (OH) D_3_ and FSH (30). However, we found no correlation between them. 

VD has been found to be related with the activation of key enzymes in steroidogenesis such as 3-beta-hydroxysteroid dehydrogenase, and it has been shown to induce the production of progesterone that consequently leads to uterine quiescence (5). Thus, VD may play a protective role for ongoing pregnancies through this mechanism.

A recent randomized controlled trial by Asadi et al. (31) showed that endometrial thickness was enhanced by the administration of VD in patients undergoing Gn and IUI treatment. Another study found that higher serum and follicular fluid 25 (OH) D_3_ levels were associated with higher pregnancy rates in women undergoing IVF (32). Similarly, a Greek study group found that follicular fluid VD levels significantly correlated with the quality of embryos (18). However, the same authors suggested that excess serum and follicular fluid vitamin levels may have a detrimental effect on IVF outcomes. These findings led us to think that an optimal level of VD is necessary for ovulation, fertilization, and implantation.

The strength of this study is that our data is single-centered and reliable. The data were obtained prospectively from the patients living in the same geographical region during the same season. There is a limitation to our study; VD deficiency was wide-spread in our study population, consistent with the results of previous studies on this issue (19, 20). The similarity of the groups in terms of high prevalence of VD deficiency may have caused 25 (OH) D_3_ levels to not be distinguishable between the groups.

## Conclusion

No significant difference was observed between pregnant and nonpregnant women who underwent ovulation induction with IUI treatment with regard to serum 25 (OH) D_3_ levels. No association was found between infertility and serum 25 (OH) D_3_ levels either. Further research which compares women who have deficient and sufficient serum VD levels is warranted. 
